# Singlet Oxygen Generated by Quercetin and Gallic Acid Leads to Oxidative Fragmentation of Flavonols in Onions and Leek

**DOI:** 10.1021/acs.jafc.5c14934

**Published:** 2026-02-05

**Authors:** Vanessa K. Fokuhl, Lea M. Kahl, Niels Heise, Marcus A. Glomb

**Affiliations:** † Institute of Chemistry, Food Chemistry, Martin-Luther-University Halle-Wittenberg, Kurt-Mothes-Str. 2, 06120 Halle/Saale, Germany; ‡ Institute of Chemistry, Organic Chemistry, Martin-Luther-University Halle-Wittenberg, Kurt-Mothes-Str. 2, 06120 Halle/Saale, Germany

**Keywords:** flavonol, oxidative fragmentation, singlet oxygen, onion, leek

## Abstract

For the first time, singlet oxygen was shown to be generated by quercetin and gallic acid under mild conditions (37 °C, pH 7) and exclusion of light. In contrast to kaempferol, quercetin induced its own oxidative fragmentation, yielding protocatechuic acid (3,4-dihydroxybenzoic acid) and 2,4,6-trihydroxybenzoic acid as the corresponding counterparts. If a 4-molar excess of gallic acid was coincubated, quercetin fragmentation reached 25 mol %, but now also kaempferol gave 7 mol % *p*-hydroxybenzoic acid. The hydroxybenzoic acids formed always showed the B-ring substitution pattern of the precursor flavonol. This reactive oxygen chemical quenching mechanism initiated by pericyclic cycloadditions was underlined by the use of singlet oxygen released from endoperoxides. Isolation and characterization of a quercetin–methanol solvent adduct pointed toward the parallel occurrence of physical quenching. The importance of singlet oxygen-mediated flavonol degradation in food matrices was verified by the detection of the fragmentation products in minced onion and leek samples.

## Graphical abstract

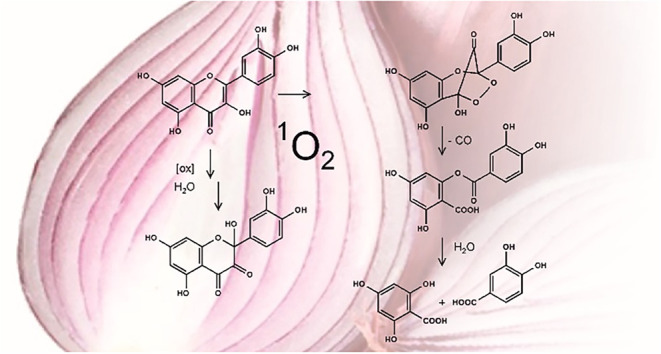

## Introduction

Flavonols have attracted considerable interest during the last decades due to their antioxidative,^1^ anticarcinogenic,^2^ antimutagenic,^3^ and anti-inflammatory^4^ properties. Therefore, they are promising therapeutic agents to treat different kinds of disorders such as neurodegenerative^5^ or cardiovascular^6^ complications. Flavonols are synthesized by plants as secondary metabolites for protection against different kinds of physiological stress, including ultraviolet (UV) radiation, pathogens or climate changes.^7^ Plants that contain high amounts of flavonols are for example *Allium* species.^8^ Composition varies between different species, with the most occurring flavonols being quercetin, kaempferol, myricetin, and isorhamnetin, usually found as *O*-glycosides.^9^ Typical glycosylation patterns are 3-*O*-monoglycosides, 4′-*O*-monoglycosides, or 3,4′-*O*-diglycosides with glucose as the most common sugar moiety.^10^ Aglycones are only present in negligible amounts but are released from glycosides when plant tissue is damaged, for example, during food processing.^11^ Quercetin has been shown to exhibit higher antioxidant activity than its glycoside rutin or other flavonols such as kaempferol.^12^ Despite their known antioxidative character, recently a prooxidative facet has gained attention.^13^ In our previous studies we were able to verify singlet oxygen generation by aspalathin and ascorbic acid even in absence of light using high-performance liquid chromatography (HPLC)-(+)-APCI-MS^2^ experiments.^14^ Singlet oxygen was then shown to initiate an oxidative rearrangement and fragmentation of dihydrochalcones, aspalathin, phloridzin, and the sweeteners naringin-dihydrochalcone and neohesperidin-dihydrochalcone to give the corresponding dihydrocinnamic acids. Among dihydrochalcones, singlet oxygen was shown to be generated only by aspalathin, and the effect was ascribed to the B-ring catechol moiety. It was therefore concluded that other flavonoids with a catechol-substituted B-ring, such as quercetin, might have similar properties. To our knowledge, the endogenous generation of singlet oxygen by flavonols under mild temperature and pH conditions and exclusion of light has yet not been demonstrated. In literature, quercetin was reported to be degraded easily by exogenous singlet oxygen to give esters and benzoic acids as the main fragmentation products.^12,15^ However, in all cases, singlet oxygen was generated photochemically.^12,15,16^ Thus, the aim of this study was to characterize flavonols for their ability to generate singlet oxygen and induce oxidative fragmentation and to show that this reaction proceeds under mild conditions in foods. Quercetin was found to be a potent candidate to give protocatechuic acid and 2,4,6-trihydroxybenzoic acid. A fragmentation mechanism was proposed and was in line with a concomitant isolated solvent adduct. Analyses of processed onions and leek underlined their importance in commonly consumed foods.

## Materials and Methods

### Chemicals

All chemicals of the highest quality available were obtained from Sigma-Aldrich (Munich/Steinheim, Germany), Roth (Karlsruhe, Germany), ACROS Organics (Geel, Belgium), Merck (Darmstadt, Germany), Fluka (Taufkirchen, Germany), and VWR Chemicals (Darmstadt, Germany), unless otherwise indicated. For all experiments, ultrapure water (Ultra Clear, Siemens, Munich, Germany) was used.

### Synthesis of Methyl Esters

Benzoic or dihydrocinnamic acids were methylated by dissolving 0.5 mg of acid in 1 mL of methanol with the addition of 100 μL of thionyl chloride. The solution was stirred for 3 h under reflux for quantitative conversion. The solvent was evaporated under reduced pressure. The purity was assessed after trimethylsilylation by gas chromatography-flame ionization detection (GC-FID) and coupled gas chromatography–mass spectrometry (GC-MS) as described below: *p*-dihydrocoumaric acid methyl ester (*m*/*z* 252, M^+·^, 1× silylated, 60%), 237 (8%), 222 (5%), 192 (15%), 179 (100%), 163 (30%), 131 (14%), 107 (12%), 89 (32%), 73 (45%); protocatechuic acid methyl ester (*m*/*z* 312, M^+·^, 2× silylated, 50%), 281 (6%), 224 (4%), 208 (4%), 193 (100%), 165 (20%), 137 (5%), 73 (50%).

### Synthesis of 1,4-Dimethylnaphthalene-1,4-endoperoxide (DMN-EP)

DMN-EP was synthesized as previously reported by Heymann based on photochemical singlet oxygen production in the presence of methylene blue as a sensitizer.^17^ Before use, the composition of the reaction products was surveyed via 1H-NMR. NMR data matched literature data reported by Wasserman.^18^ DMN-EP synthesis gave a mixture of about (1:1) consisting of DMN-EP and DMN. Singlet oxygen was released by 50% within 55 min and fully within 6 h of incubation at 37 °C.^14^

### Aerated Phloridzin Incubations

Phloridzin (0.5 mM) was dissolved in a 1:1 mixture of methanol and phosphate buffer (0.1 M, pH 7) and incubated in screw cap vials. Incubations were kept in a shaker at 37 °C under exclusion of light for 24 h. To a part of the samples, 2 mM of a second phenol (benzoic acid, *p*-hydroxybenzoic acid, protocatechuic acid, vanillic acid, syringic acid, gallic acid, cinnamic acid, *p*-coumaric acid, caffeic acid, ferulic acid, sinapic acid, aspalathin, catechin, epigallocatechin gallate, quercetin, kaempferol, DMN-EP) was added to test for a singlet oxygen-generating substance. Incubations of phloridzin with epigallocatechin gallate and gallic acid were repeated in phosphate buffer (0.1 M, pH 7) and shown to give the same fragmentation rates. The formation of *p*-dihydrocoumaric acid was analyzed by GC-FID and GC-MS after silylation. Sample preparation was as follows: For GC analysis, a 1 mL aliquot of the sample was acidified with 1 mL of 6 M HCl, extracted twice with 2 mL of diethyl ether for quantitation of *p*-dihydrocoumaric acid, or extracted with ethyl acetate for quantitation of other phenolic acids, and the solvent was removed under an argon atmosphere. The dried extracts were dissolved in 50 μL of pyridine, and 50 μL of *N*,*O*-bis­(trimethylsilyl)­acetamide with 5% trimethylchlorosilane were added. Samples were kept at room temperature for 1 h prior to being injected into the GC system.

### Detection of Singlet Oxygen in Aerated Phloridzin Incubations

The above reactions for quercetin and gallic acid were performed in the presence of 9,10-diphenylanthracene (DPA, 0.1 mM) in a 1:1 mixture of phosphate buffer (0.1 M, pH 7) with acetonitrile according to previous works.^14^ Singlet oxygen DPA-endoperoxide was analyzed by LC-MS.

### Aerated Polyphenol Incubations

Polyphenols (0.5 mM; quercetin, isoquercitrin, rutin, fisetin, rhamnetin, isorhamnetin, kaempferol, and apigenin) were dissolved in a 1:1 mixture of methanol and phosphate buffer (0.1 M, pH 7) and incubated in screw cap vials. Incubations were kept in a shaker at 37 °C under the exclusion of light for 24 h. To a part of the samples, 2 mM of gallic acid or DMN-EP was added as a singlet oxygen-generating substance at the start of incubation. The formation of fragmentation products was analyzed by GC-FID and GC-MS after silylation, as described above. Flavonols were analyzed by using high-performance liquid chromatography with diode array detection (HPLC-DAD) and coupled high-performance liquid chromatography–mass spectrometry (HPLC-MS). For HPLC analysis, an aliquot of the incubations was directly injected.

### Aerated Onion/Leek Incubations

Red onions and leek were purchased from a local food store. Edible parts were shredded, and 8 g was suspended in 10 mL of methanol/water (1:1 v/v). Samples were incubated in an Erlenmeyer flask, kept in a shaker at 37 °C under exclusion of light, and worked up in the same way as the polyphenol incubations. For HPLC analysis, samples were centrifuged, and the supernatant was used for analysis.

### Deaerated Polyphenol/Onion/Leek Incubations

The incubations were modified by adding 1 mM diethylenetriaminepentaacetic acid to the phosphate buffer or water. All solvents were degassed in an ultrasonic bath for 15 min and with helium for 20 min. Samples were incubated in flared vials without air for polyphenol incubations and in an Erlenmeyer flask under an argon atmosphere for onion and leek incubations.

### Isolation of Quercetin–Methanol Adduct

For isolation of the quercetin–methanol adduct, the incubation of 0.5 mM quercetin with 2 mM gallic acid was upscaled to 4 L and incubated for 6 h. Solvents were evaporated under reduced pressure at 30 °C and the solid was resolved in 20 mL of water and preseparated by flash chromatography (RP18, 40–63 μm, methanol/water (1/1, v/v)), fractions containing the methanol adduct combined and evaporated, and the final residue taken up in 2 mL methanol/water (3/7, v/v) with 0.8 μL mL^–1^ formic acid for final preparative reversed-phase chromatography. Chromatographic fractions were monitored by an HPLC-DAD.

### Preparative Reversed-Phase Chromatography

The glass column (Merck, LOBAR LiChroprep RP-18 (31.0 cm × 2.5 cm, 40–63 μm), Darmstadt, Germany) was connected to a Waters 510 HPLC-pump (Guyancourt, France) and a Gynkotek SP-6 UV-detector (Germering, Germany), operating at 280 nm and 5 mL min^–1^. Eluted liquids were collected in fractions of 10 mL with a fraction collector (Labomatic, Labocol Vario 4000, Allschwil, Switzerland). Chromatograms were recorded on a plotter (Shimadzu, C-R6A Chromatopac, Duisburg, Deutschland). 1 mL of the dissolved sample was injected for a run. Separations were run with isocratic eluents of methanol/water (3/7, v/v) with the addition of 0.8 mL L^–1^ formic acid. Target products **2** and **3** eluted between *t*_R_ = 110–180 min. The solvent of the collected fractions was evaporated under reduced pressure at 30 °C. The products were yielded as white, amorphous solids and stored at 4 °C.

### High-Performance Liquid Chromatography – Diode Array Detection (HPLC–DAD)

For polyphenol and onion/leek analyses, a Jasco PU-2080 Plus quaternary gradient pump with a degasser (DG2080–54), quaternary gradient mixer (LG 2080–02), multiwavelength detector (MD-2015 Plus) (Jasco, Gross-Umstadt, Germany), Waters 717 plus autosampler, and column oven (Techlab Jet Stream np K-3, Erkerode, Germany) was used. Chromatographic separations were performed on stainless steel columns (Vydac CRT, 201TP54, 250 × 4.6 mm; RP-18, 5 μm; Hesperia, CA) using a flow rate of 1.0 mL min^–1^. The column temperature was always 22 °C. The mobile phase consisted of water (solvent A) and MeOH (solvent B), and to both solvents (A and B), 0.8 mL L^–1^ formic acid was added. Samples were analyzed using a gradient system: samples were injected at 10% B. The gradient was changed linearly to 23.5% B in 20 min, to 60% B after 20 min, to 72.1% B after 10 min, and then to 100% B after 2 min and held for 6 min. The gradient was changed linearly back to 10% B in 2 min and held for 10 min. The effluent was monitored at 254, 280, and 370 nm. For quantitation, an external calibration based on standard solutions of authentic references dissolved in the same solvent as the sample was used.

### Coupled High-Performance Liquid Chromatography – Mass Spectrometry (HPLC-MS)

For HPLC-MS a Jasco PU-2080 Plus quaternary gradient pump with a degasser (DG-2080–54), quaternary gradient mixer (LG 2080–04) (Jasco, Gross-Umstadt, Germany), AS-2057 Plus autosampler set at 4 °C and column oven (Jasco Jetstream II) set at 22 °C was used. Chromatographic separations were performed on a stainless steel column (Vydac CRT, 201TP54, 250 mm × 4.6 mm; RP-18, 5 μm; Hesperia, CA). A flow rate of 1.0 mL min^–1^ was used. The column temperature was always 22 °C. The mobile phase consisted of water (solvent A) and MeOH (solvent B), and to both solvents (A and B), 0.8 mL L^–1^ formic acid was added. Samples were analyzed using a gradient system: samples were injected at 25% B. The gradient was changed linearly to 65% B in 9 min, then to 100% B in 5 min, and held for 2.8 min. The gradient was changed linearly back to 25% B in 0.2 min and held for 3 min. The effluent was monitored at 280 nm. Mass analyses were conducted on an API 4000 QTrap LC-MS/MS system (AB Sciex, Concord, ON, Canada) equipped with a turbo ion spray source using electrospray ionization (ESI) in negative mode for full-scan analysis: sprayer capillary voltage of −4.5 kV, nebulizing gas flow of 70 mL min^–1^, heating gas of 80 mL min^–1^ at 650 °C, curtain gas of 40 mL min^–1^, declustering potential −30 V and entrance potential of −10 V.

### Gas Chromatography – Flame Ionization Detection (GC-FID)

A Nexis GC-2030 gas chromatograph (Shimadzu, Duisburg, Germany) equipped with an autosampler (AOC-20 Plus Series) and an FID was used with helium 4.6 as a carrier gas in constant-flow mode (linear velocity of 25.2 cm/s and flow of 1.0 mL/min). Samples (1 μL) were injected to a split–splitless injector at 220 °C (Split ratio of 19) and separated on a HP-5 capillary column (30 m × 0.32 mm × 0.25 μm, Agilent Technologies, Santa Clara, CA). The detector was set at 300 °C. The GC oven temperature was started at 80 °C, raised to 200 °C (8 K/min) and then to 270 °C (10 K/min), and held for 10 min. The total run time was 32 min. Retention times after silylation: *p*-hydroxybenzoic acid, *t*_R_: 14.9 min; *p*-dihydrocoumaric acid methyl ester, *t*_R_: 15.0 min; protocatechuic acid methyl ester, *t*_R_: 16.2 min; *trans*-aconitic acid, *t*_R_: 16.4 min; *p*-dihydrocoumaric acid, *t*_R_: 16.6 min; vanillic acid, *t*_R_: 16.7 min; protocatechuic acid, *t*_R_: 17.4 min; gallic acid, *t*_R_: 18.8 min; 2,4,6-trihydroxybenzoic acid, *t*_R_: 19.2 min. For quantitation, an external calibration based on standard solutions of authentic references was used.

### Coupled Gas Chromatography – Mass Spectrometry (GC-MS)

A Thermo Finnigan Trace GC Ultra coupled to a Thermo Finnigan Trace DSQ (Thermo Fisher Scientific GmbH, Dreieich, Germany) was used with 5.0 helium atoms as a carrier gas in constant-flow mode (linear velocity of 35.0 cm/s). Samples (1 μL) were injected to a split–splitless injector at 220 °C (split ratio of 19) and separated on a DB-5MS capillary column (30 m × 0.25 mm × 0.25 μm + 10 m Guard, Agilent Technologies, Santa Clara, CA). MS conditions were as follows: 70 eV with electron-impact ionization (source temperature of 230 °C and emission current of 80 mA) in full-scan mode (mass range of *m*/*z* 50–650). The oven temperature program was identical to that of GC-FID. Retention times after silylation: *p*-hydroxybenzoic acid, *t*_R_: 14.9 min; *p*-dihydrocoumaric acid methyl ester, *t*_R_: 15.0 min; protocatechuic acid methyl ester, *t*_R_: 16.1 min; *trans*-aconitic acid, *t*_R_: 16.5 min; *p*-dihydrocoumaric acid, *t*_R_: 16.7 min; vanillic acid, *t*_R_: 16.7 min; protocatechuic acid, *t*_R_: 17.4 min; gallic acid, *t*_R_: 19.0 min; 2,4,6-trihydroxybenzoic acid, *t*_R_: 19.2 min.

### High-Resolution Mass Determination (HR-MS)

Negative-ion high resolution electrospray ionization (ESI) mass spectra were obtained from a TripleToF 6600–1 mass spectrometer (Sciex, Darmstadt, Germany) equipped with a heated ESI-DuoSpray ion source and was controlled by Analyst 1.7.1 TF software (Sciex). The ESI source operation parameters were as follows: ion spray voltage 3.7 kV; nebulizing gas: 60 psi; source temperature, 450 °C; drying gas: 70 psi; curtain gas: 35 psi. Data acquisition was performed in the MS^1^-ToF mode, scanned from 100 to 1500 Da with an accumulation time of 50 ms.

### Nuclear Magnetic Resonance Spectroscopy (NMR)

One- and two-dimensional NMR-experiments (^1^H, ^2^H, ^13^C, HMBC, HSQC) were performed on a Jeol-ECZL600G at 300 K. The signal of the residual protons of the deuterated solvent was used as an internal reference for 1H-NMR, and the solvent peak of the deuterated solvent was used as an internal reference for 13C-NMR, respectively (referenced to TMS).

### Statistical Analysis

Quantitations were performed in triplicates. The limit of detection (LOD) and limit of quantitation (LOQ) were calculated at signal-to-noise ratios of 3 and 10, respectively. The values for carboxylic acid fragments are given as mol % from phloridzin (*p*-dihydrocoumaric acid: 0.03/0.09%) or from the respective flavonol (protochatechuic acid: 0.01/0.04%; *p*-hydroxybenzoic acid: 0.02/0.06%; vanillic acid: 0.03/0.09%).

## Results and Discussion

### Singlet Oxygen-Triggered Degradation of Phloridzin

To gain deeper insight into the reaction of singlet oxygen with flavonoids and to extend the spectrum of phenolic compounds that can generate singlet oxygen, our established model of oxidative phloridzin degradation was used (Figure SI-1). Originally developed for the dihydrochalcone asphalatin, here it was extended to different flavonoids, cinnamic acids, and benzoic acids (Table 1).^14,19^ After 24 h of incubation under mild conditions (37 °C, pH 7, aeration), *p*-dihydrocoumaric acid, as the singlet oxygen-induced fragmentation product of phloridzin, was quantitated at 1.83 mol % with asphalatin, while phloridzin alone was stable. Importantly, photochemical singlet oxygen generation was ruled out, as all incubations were conducted in the dark. This was in line with our previous results, where one structural element required for singlet oxygen generation was shown to be the catechol moiety located at the B-ring, and thus, structures with no or single phenolic groups or hydroquinone methyl ether moieties, such as vanillic acid or ferulic acid, did not induce fragmentation. In contrast, compounds with catechol groups, such as protocatechuic acid, caffeic acid, catechin, and quercetin, gave fragmentation yields ranging from 0.54 to 1.58 mol %. However, the most effective singlet oxygen sources were gallic acid and epigallocatechin gallate with 3.15 and 3.24 mol % phloridzin scission, respectively, with pyrogallol/gallo-hydroxylation motives. As gallic acid is by far the more frequent plant and food constituent, it was chosen for all follow-up experiments. It has to be mentioned that, because of the poor solubility of some flavonoids, incubations were carried out in a methanol-phosphate buffer mixture. However, it was confirmed with the more soluble representatives that methanol did not interfere with the fragmentation reaction, giving virtually the same yields as incubated solely in phosphate buffer. Phloridzin fragmentation to *p*-dihydrocoumaric acid is a specific but indirect measure of the presence of singlet oxygen. Thus, direct identification was performed with 9,10-diphenylanthracene as a selective trapping probe. Figure 1 shows exemplary results for quercetin and gallic acid, demonstrating that the resulting endoperoxides were virtually identical to an authentic reference standard.

**Figure 1:**
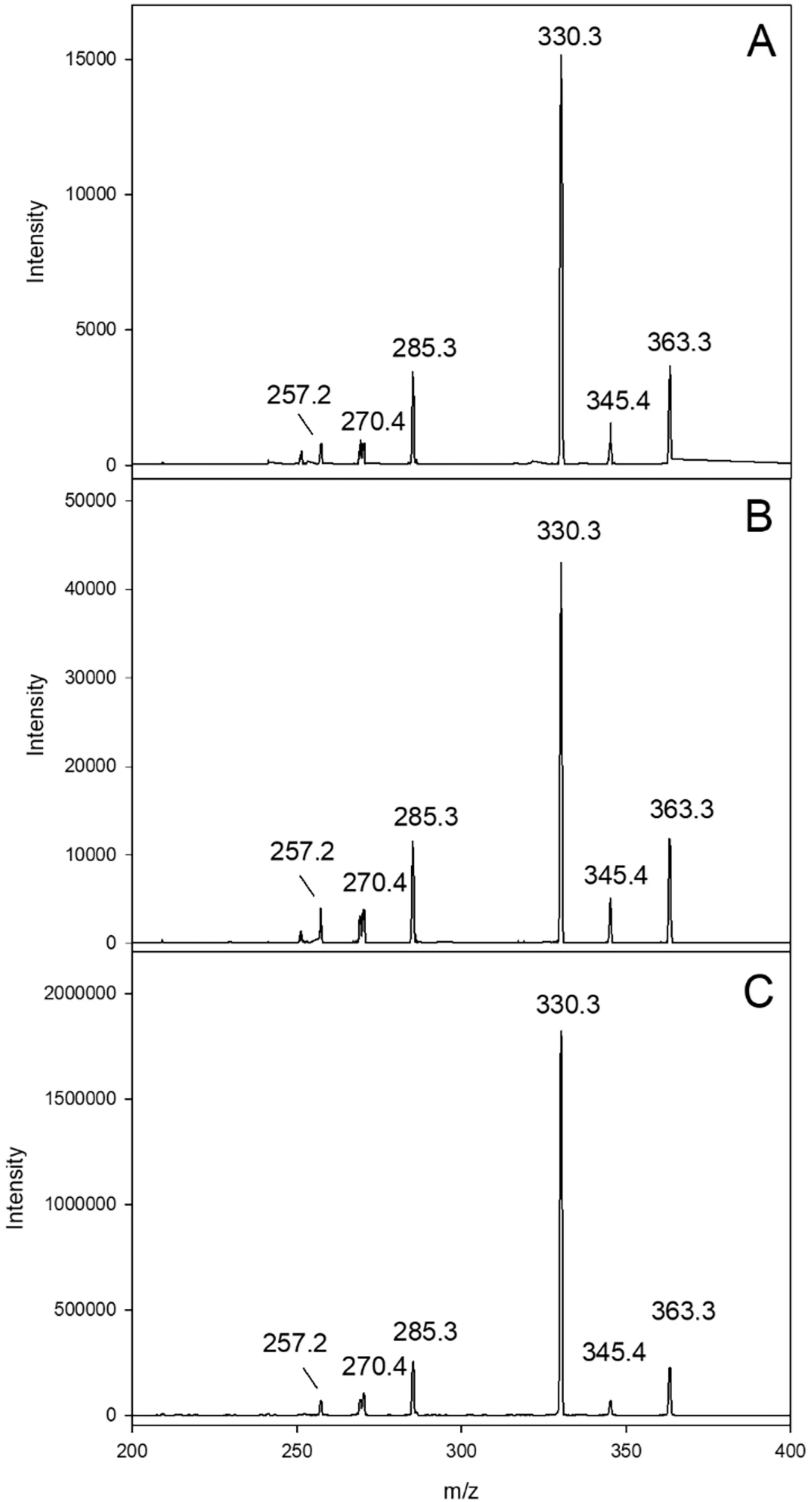
Formation of specific singlet oxygen endoperoxides in quercetin and gallic acid incubations with phloridzin in the presence of 9,10-diphenylanthracene (DPA) (37 °C, pH 7, aeration). Verification of DPA-endoperoxide by collision-induced dissociation (CID) of *m*/*z* 363 (M + H)+ via HPLC­(+)-APCI-MS2, (A) quercetin incubation; (B) gallic acid incubation; (C) authentic reference standard.

**Table 1:** Formation of *p*-Dihydrocoumaric Acid from 0.5 mM Phloridzin Triggered by 2 mM of Different Phenolic Compounds (24 h, 37 °C, pH 7, Aeration, Exclusion of Light)

	*p*-dihydrocoumaric acid [mol-%]
benzoic acids	
benzoic acid	<LOD
*p*-hydroxybenzoic acid	<LOD
protocatechuic acid	0.54 ± 0.01%
vanillic acid	<LOD
syringic acid	<LOD
gallic acid	3.15 ± 0.04%
cinnamic acids	
cinnamic acid	<LOD
*p*-coumaric acid	<LOD
caffeic acid	1.58 ± 0.15%
ferulic acid	<LOD
sinapic acid	<LOD
flavonoids	
aspalathin	1.83 ± 0.03%
catechin	1.26 ± 0.07%
epigallocatechin gallate	3.24 ± 0.15%
quercetin	0.56 ± 0.02%
kaempferol	<LOD

### Singlet Oxygen-Triggered Degradation of Quercetin and Kaempferol

Flavon-3-ols have been reported to be highly unstable in the presence of photochemically generated singlet oxygen, but also when exposed to atmospheric oxygen, and hydroxybenzoic acids were identified as the degradation products.^12,20^ Here, we first focused on the degradation of quercetin and kaempferol by gallic acid-induced singlet oxygen, as they are the most abundant flavonoids in vegetables, typically in the glycosidic form, with quercetin quantitatively exceeding kaempferol.^21^ Again, experiments were performed under mild conditions and in the dark (Figure 2). In the presence of gallic acid, quercetin was totally eliminated after 4 h of incubation, while protocatechuic acid accumulated to 25 mol-% at 24 h. This discrepancy clearly shows that singlet oxygen-induced fragmentation is an important mechanistic pathway, but other reactions like oxidative coupling must occur in parallel. Notably, a clear but much slower degradation of quercetin (−32 mol-% at 24 h) occurred together with a moderate protocatechuic acid formation (7 mol-%) even in the absence of gallic acid. This underlined the above results that quercetin alone can intrinsically generate singlet oxygen under aeration to induce its own degradation. It can also be seen that even at 0 h, quercetin never started at 100 mol-%. This was inline with other reports that under aeration quercetin decomposition already proceeds during sample preparation.^20^ On the other hand, kaempferol showed almost no degradation within the 24 h in the absence of gallic acid, and no *p-*hydroxybenzoic acid was monitored. This was expected as kaempferol lacks any catechol or gallomotives and thus cannot generate singlet oxygen intrinsically. In the presence of gallic acid, oxidative scission was induced, resulting in complete breakdown within 16 h of incubation, much slower compared to quercetin reactions. In addition, the singlet oxygen-induced fragmentation product *p*-hydroxybenzoic acid showed up with about 7 mol-%. Interestingly, in all reactions with gallic acid, another carboxylic acid was identified as *t*-aconitic acid in comparison to a commercial standard. Indeed, this gallic acid degradation product has been reported to be induced by enzymes or under strong alkaline or oxidative conditions.^22–24^ However, this is not the focus of the present investigation.

**Figure 2:**
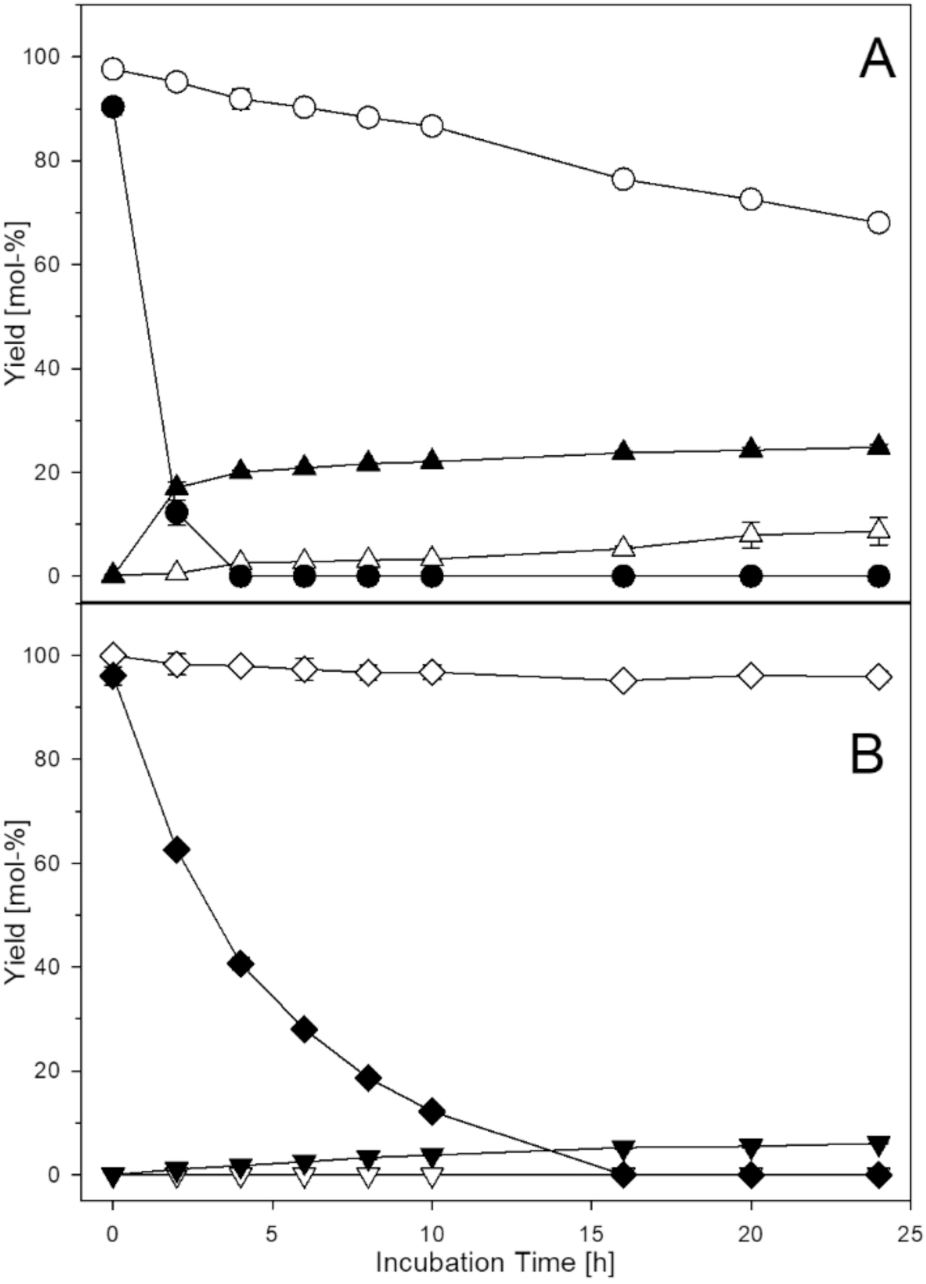
Fragmentation of 0.5 mM flavon-3-ols in the presence or absence of 2 mM gallic acid (37 °C, pH 7, aeration). (A) quercetin without (○) and with gallic acid (●), protocatechuic acid without (Δ) and with gallic acid (▲). (B) kaempferol without (◊) and with gallic acid (⧫), *p*-hydroxybenzoic acid without (∇) and with gallic acid (▼).

### Mechanistic Aspects of Singlet Oxygen-Induced Flavon-3-ol Degradation

For clarification of the fragmentation mechanism, experiments with gallic acid were extended to other frequent flavonols with different substitution patterns (Table 2). Tournaire et al. proposed a mechanism for photochemically generated singlet oxygen, inducing the fragmentation of flavonols in absolute methanol.^12^ Starting with a [2 + 2] cycloaddition of singlet oxygen and the 2,3-C-ring double bond to give highly unstable dioxetanes, degradation was proposed via ring opening and loss of carbon monoxide to give an ester of 2,4,6-trihydroxybenzoic acid from the former A-ring with a hydroxybenzoic acid from the former B-ring (Figure 3, dashed arrows). That ester from quercetin was isolated and characterized. Focusing on the antioxidative activity, they concluded that the physical quenching is controlled by the B-ring substitution, while the chemical quenching (*i.e*., the reactivity toward singlet oxygen) depends on the C-ring structure. This is in contrast to the aqueous incubation conditions used in the present investigations, as no such ester structures were found by coupled liquid chromatography mass spectrometry (LC-MS). Obviously, these esters are highly susceptible to hydrolysis, as fragmentation always led to free 2,4,6-trihydroxybenzoic acid, as exemplified by Figure 4 for quercetin incubations in comparison to an authentic reference standard. After derivatization, coupled gas chromatography–mass spectrometry (GC-MS) showed *m*/*z* 443 as the M-15, typical for multiple trimethylsilylated structures. As a highly reactive intermediate, 2,4,6-trihydroxybenzoic acid was not quantitated. However, in line with the literature, the carboxylic acid counterpart always showed the former B-ring substitution, *e.g*., isorhamnetin led to vanillic acid. Interestingly, in parallel to the ester hydrolysis, the methyl ester was detected. Although an artifact of the water–methanol solvent used, this verified the existence of an ester intermediate within the singlet oxygen-mediated flavonol scission. Figure 5 depicts the identification of the protocatechuic acid methyl ester in quercetin-gallic acid incubations. *M/z* 312 denotes the trimethylsilylated molecular ion peak, *m*/*z* 297 (−15) typical methyl fragmentation of these silylethers, and *m*/*z* 281 (−31) typical elimination of OCH3 from methyl esters. To exclude other formation pathways during the oxidative breakdown, methanol was exchanged for ethanol, and the respective ethanol ester structures were found (not shown). Consequently, the quantitative yields given in Table 2 are always the sum of concomitantly formed free and methylated phenolic acids. It has to be noted that the formation of the artifact methyl esters strongly depended on the flavon-3-ol species fragmented. While precursors giving protocatechuic acid showed a ratio of 40% methyl ester formation, formation of *p*-hydroxybenzoic acid was only accompanied by about 1% of the methyl ester, while vanillic acid formation never entailed any ester formation.

**Figure 3:**
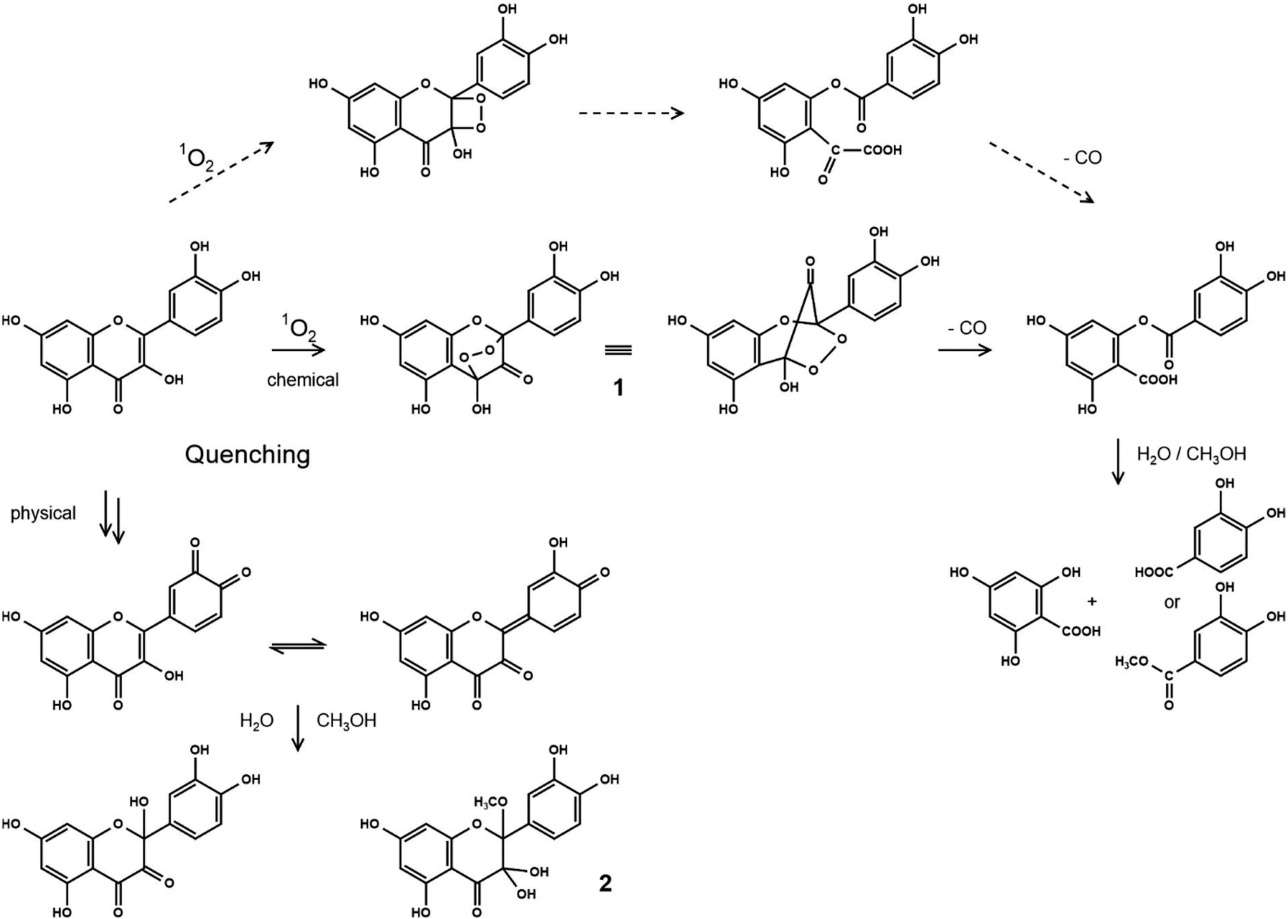
Proposed mechanism of quercetin degradation in the presence of singlet oxygen under aqueous mild conditions in the absence of light: formation of methanol adduct **2** (physical quenching) and fragmentation (chemical quenching) via bicyclic endoperoxide **1** to give protocatechuic acid with its methyl ester and 2,4,6-trihydroxybenzoic acid. The fragmentation via a dioxetane intermediate as proposed by Tournaire et al.^12^ is outlined by dashed arrows.

**Figure 4:**
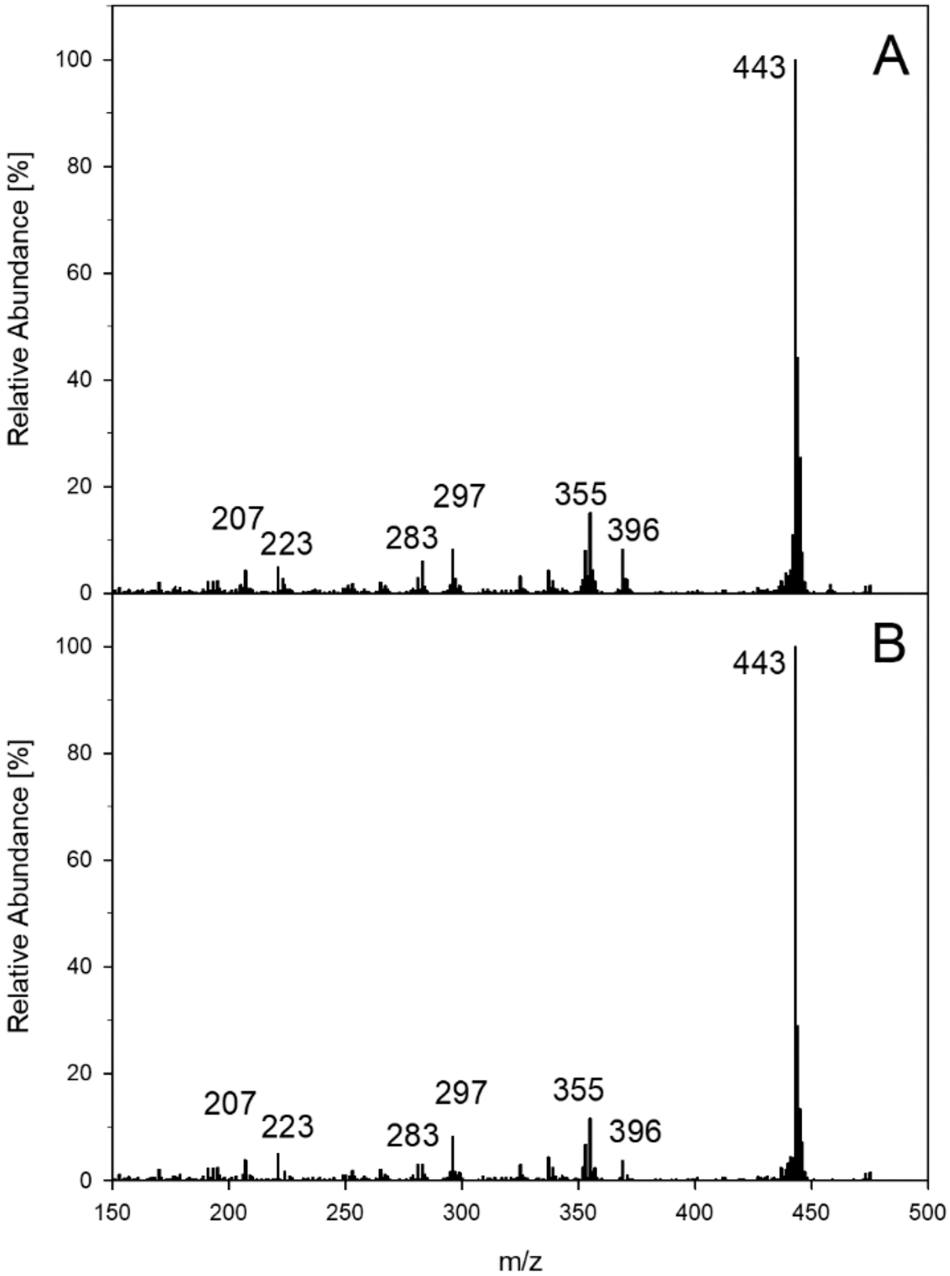
Verification of 2,4,6-trihydroxybenzoic acid in incubations of 0.5 mM quercetin with 2 mM gallic acid (37 °C, pH 7, aeration). GC-MS after trimethylsilylation; (A) authentic reference standard, (B) sample workup.

**Figure 5:**
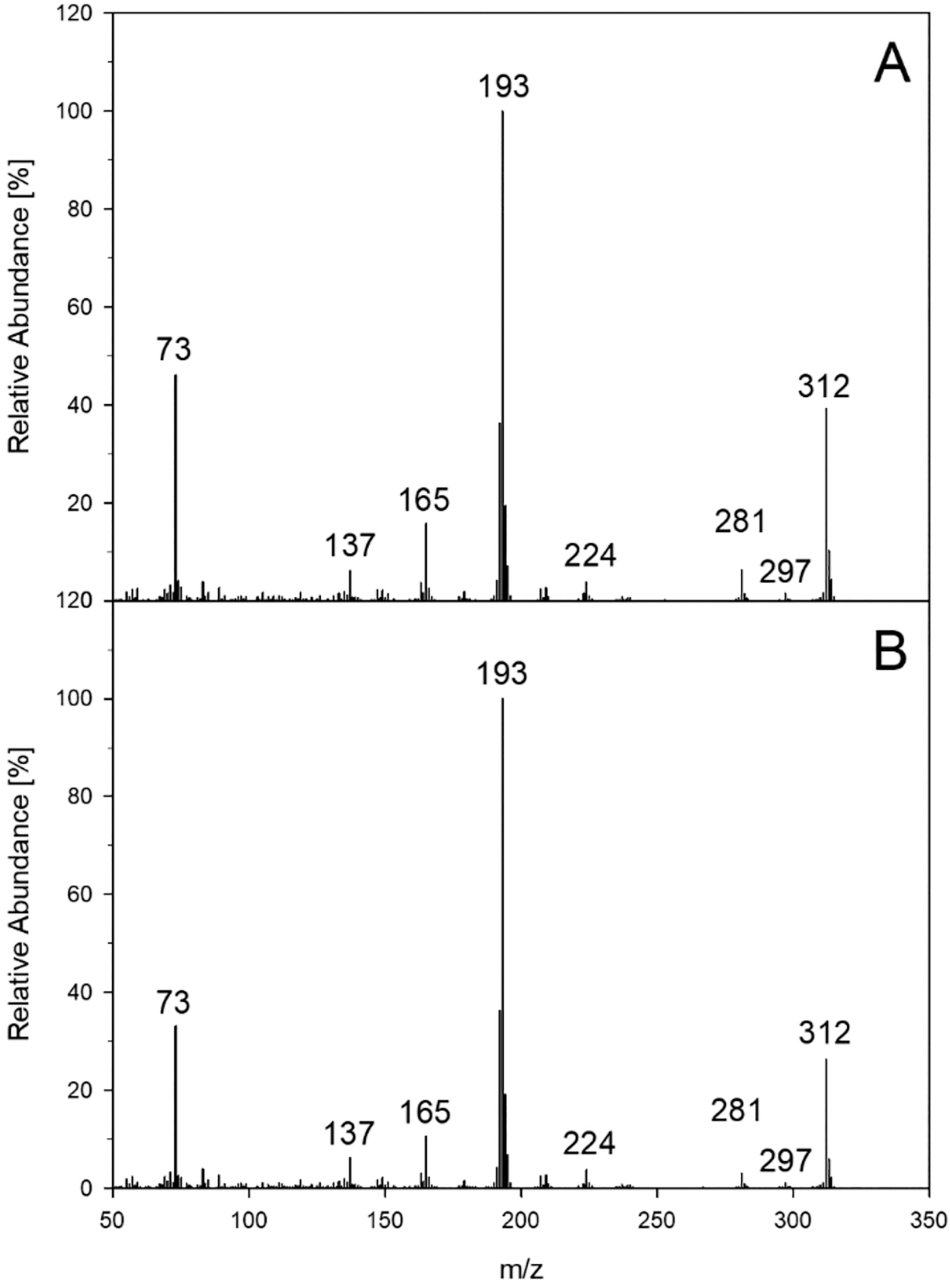
Verification of protocatechuic acid methyl ester in incubations of 0.5 mM quercetin with 2 mM gallic acid (37 °C, pH 7, aeration). GC-MS after trimethylsilylation; (A): authentic reference standard, (B): sample workup.

**Table 2:** Fragmentation of Different Flavonols (0.5 mM) Induced by 2 mM Gallic Acid or DMN-EP as Singlet Oxygen Source (24 h, 37 °C, pH 7, Exclusion of Light)^a^

	fragmentation	
	yield [mol %]	product
*aerated with gallic acid*		
quercetin	24.6 ± 1.1%	PCA
isoquercitrin	1.9 ± 0.4%	PCA
rutin	1.3 ± 0.12%	PCA
fisetin	31.6 ± 0.4%	PCA
rhamnetin	18.2 ± 0.9%	PCA
isorhamnetin	15.5 ± 0.8%	VA
kaempferol	6.8 ± 0.4%	HBA
apigenin	<LOD	(HBA)
*deaerated with gallic acid*		
quercetin	5.9 ± 0.4%	PCA
*deaerated with DMN-EP*		
quercetin	18.2 ± 1.6%	PCA
kaempferol	4.7 ± 0.4%	HBA

a PCA: protocatechuic acid; VA: vanillic acid; HBA: *p*-hydroxybenzoic acid.

Table 2 supports the notion that the proposed initial cycloaddition to give an endoperoxide structure is strongly controlled by the electron density of the 2,3-unsaturated C-ring. A free 3-OH function, but also B-ring catechol or hydroquinone ether moieties, always led to high fragmentation yields. As expected, the +M effects are obviously prerequisite for the reaction with the electrophilic singlet oxygen to induce cycloaddition. This is prominent for the flavone apigenin with no 3-OH substitution, giving no fragmentation, for kaempferol with single B-ring hydroxylation with moderate fragmentation (∼7 mol %), but also for 3-*O*-glycosilated quercetin derivatives isoquercitrin and rutin with low yields (∼1–2 mol %). For the latter also, steric hindrances were discussed.^12,25^ Change of A-ring substitution as with fisetin (lack of 5-OH) or rhamnetin (7-OCH_3_) did not change reactivity.^26^ To pinpoint the need for singlet oxygen in the reaction, quercetin-gallic acid reactions were first performed under deaeration to give significantly lower fragmentation yields and then under deaeration with dimethylnaphthalene-endoperoxide (DMN-EP) instead of gallic acid for *in situ* generation of singlet oxygen under strict exclusion of light to almost reach the protocatechuic/*p*-hydroxybenzoic acid levels of aerated quercetin/kaempferol gallic acid incubations. DMN-EP was shown to quantitatively release singlet oxygen in aqueous media within a short time.^14^

The fragmentation of flavon-3-ols in the presence of singlet oxygen represents a mechanism of chemical quenching of reactive oxygen species. Above hypothesized [2 + 2] cycloaddition to give a dioxetane does not explain loss of the C-3 carbon function via an intermediate ester bearing an α-oxocarboxylic acid moiety to give carbon monoxide (Figure 3, dashed arrows).^12^ Alternatively, an addition of singlet oxygen to C-2 and C-4 of the C-ring was proposed, leading to a five-membered endoperoxide **1** (Figure 3, solid arrows).^27^ This cycloaddition must involve a concomitant hydrogen transfer between the 3-OH and C-4 carbonyl function, which is highly likely due to the known hydrogen chelating effect between C-3 and C-4 of flavon-3-ols. Decomposition of such bicyclic five-membered endoperoxides has been shown to lead to carbonyl fragments under the release of carbon monoxide. 2,3,4,5-Tetraphenylcyclopentadiene gave a dibenzoylstilbene derivative upon photolysis.^28^ Indeed, after synthesis of position C-3 ^13^C-labeled quercetin, the release of carbon monoxide from this specific position was verified upon treatment with photochemically generated singlet oxygen.^29^ Nevertheless, the final ester hydrolyses to 2,4,6-trihydroxybenzoic acid and a hydroxybenzoic acid derivative or the respective methyl ester in the presence of methanol.

It has to be emphasized, that all mechanistic studies on flavonol chemistry were conducted (I) in absence of water, typically in pure methanol, and (II) under UV irradiation to induce chemical and physical quenching effects.^30^ While UV-light in the presence of oxygen led to the cycloaddition of singlet oxygen followed by fragmentation (chemical quenching), especially under limitation of oxygen, photoproducts reacted via the excited triplet state of flavonols to induce radical follow-up reactions, *e.g*., with triplet oxygen, or, more importantly, after formation of quinones, addition of nucleophiles resulted in methanol in 2-methoxyflavo-3,4-diones (physical quenching). Those approaches were totally different from the present investigation, where mild temperature and pH under exclusion of light in aqueous media were used to mimic food-related conditions. However, we were able to isolate the C-2 methanol acetalic adduct 2-methoxy-3,3,5,7,3′,4′-hexahydroxyflavanone **2** from quercetin-gallic acid reactions (Figure 3, Table 3). This structure was so far only identified from irradiated methanol solutions via LC-MS,^30^ while 2-hydroxyflavo-3,4-dione was characterized after photolysis from alkalized aqueous quercetin solutions by NMR.^31^ Thus, most resonances were comparable apart from the methoxy function (2.94 ppm, s, 3H) at C-2 with 107 ppm, and instead of a carbonyl function at C-3, hydrate formation was verified with typical 91 ppm measured herein. It has to be mentioned that adduct **2** formation was always accompanied by an equilibrium with a minor second methanol addition at C-7, giving 2,7-dimethoxy-3,3,5,3′,4′-pentahydroxyflavanone **3**, which is discussed in the Supporting Information. With LC-MS, adduct **2** formation was monitored at the highest level with flavon-3-ols that also showed the highest fragmentation rates, *i.e*., with the highest singlet oxygen-generating capacity. As no UV irradiation was used, this strongly suggested that, also under the aqueous conditions used herein, flavonols can not only chemically quench the intrinsically generated singlet oxygen, entailing fragmentation, but at the same time, this singlet oxygen is physically quenched to induce oxidation.

**Table 3:**
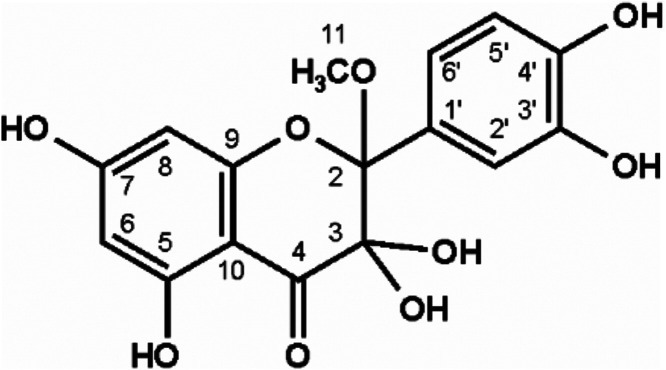
High-Resolution Mass and ^1^H- and ^13^C-NMR Spectroscopic Data of 2-Methoxy-3,3,5,7,3′,4′-hexahydroxyflavanone **2** (in DMSO-*d6*)

HR-MS [M-H]^−^ (*m/z*)		349.0560
calcd. C_17_H_15_O_9_^–^ (*m/z*)		349.0565
C/H	δ ^1^H [ppm]	δ ^13^C [ppm]
2	-	106.8
3	-	90.7
4	-	194.7
5	-	163.1
6	5.95 (s, 1H)	96.3
7	-	166.8
8	5.99 (s, 1H)	95.9
9	-	158.4
10	-	99.7
11	2.94 (s, 3H)	50.1
1′	-	124.1
2′	7.03 (d, 1H)	116.8
	^4^ *J* = 2.1 Hz	
3′	-	144.1
4′	-	145.8
5′	6.74 (d, 1H)	114.4
	^3^ *J* = 8.2 Hz	
6′	6.87 (dd, 1H)	120.3
	^3^ *J* = 8.2 Hz	
	^4^ *J* = 2.1 Hz	

### Singlet Oxygen-Induced Flavon-3-ol Degradation in Onion Samples

The relevance of singlet oxygen-mediated degradation in food was tested in commercial onion samples. Red onions were chosen for the incubations due to the high quercetin content, occurring almost exclusively as mono- and diglucosides.^10^ Quercetin aglycone was detected in minor amounts at the beginning of the incubation period (Figure 6). This is also due to the fact that the aglycone is present mainly in the outer dry paper skin of onions,^32^ which was removed before incubation, so only the edible parts were analyzed. The glucoside pattern was found to be in accordance with literature data, with quercetin-3,4′-*O*-diglucoside and quercetin-4′-*O*-monoglucoside being the most abundant. Enzymatic hydrolysis of the glucosides started immediately after shredding. Again, a water–methanol mixture was used to keep especially the flavonol aglycons in solution. Quercetin-3,4′-*O*-diglucoside showed a rapid decay within the first 4 h of incubation (233 mg was reduced to 114 mg/kg fresh wt) and was completely eliminated after 16 h. This was paralleled by an increase of quercetin-4′-*O*-monoglucoside, which reached a maximum after 6 h (178 mg/kg fresh wt) before hydrolysis prevailed to give free quercetin. Isoquercitrin was detected in very small amounts and not quantitated. Another group of small peaks showed the same pattern during hydrolysis as the quercetin derivatives. By LC-MS, these were identified qualitatively as the glucosides of isorhamnetin by comparison to an authentic reference standard. Indeed, these flavonoids are minor components in red onions.^10^ Parallel to the release of quercetin from its glucosides, protocatechuic acid (PCA) and its methyl ester (PCA-ME) were detected to reach about 4.5 mg/kg fresh wt after 24 h (Figure 7). Likewise, formation of vanillic acid derived from the oxidative fragmentation of isorhamnetin was verified, however, at much lower levels. To exclude any formation by enzymatic hydrolysis from higher molecular structures, incubations were repeated under deaeration. No detection or increase was monitored. Hence, the above-established singlet oxygen-induced fragmentation of flavonols obviously also occurs during common food technology. In case of onions, the dominating quercetin aglycon is first set free by hydrolysis after rupture of cell integrity, and then is the target for intrinsic singlet oxygen produced by the catechol moiety to induce fragmentation to protocatechuic acid, but also vanillic acid from released isorhamnetin. As isorhamnetin with a B-ring hydroquinone methyl ether moiety cannot generate singlet oxygen itself, the causal relation was proved by a quercetin-isorhamnetin coincubation to give both protocatechuic and vanillic acid.

**Figure 6:**
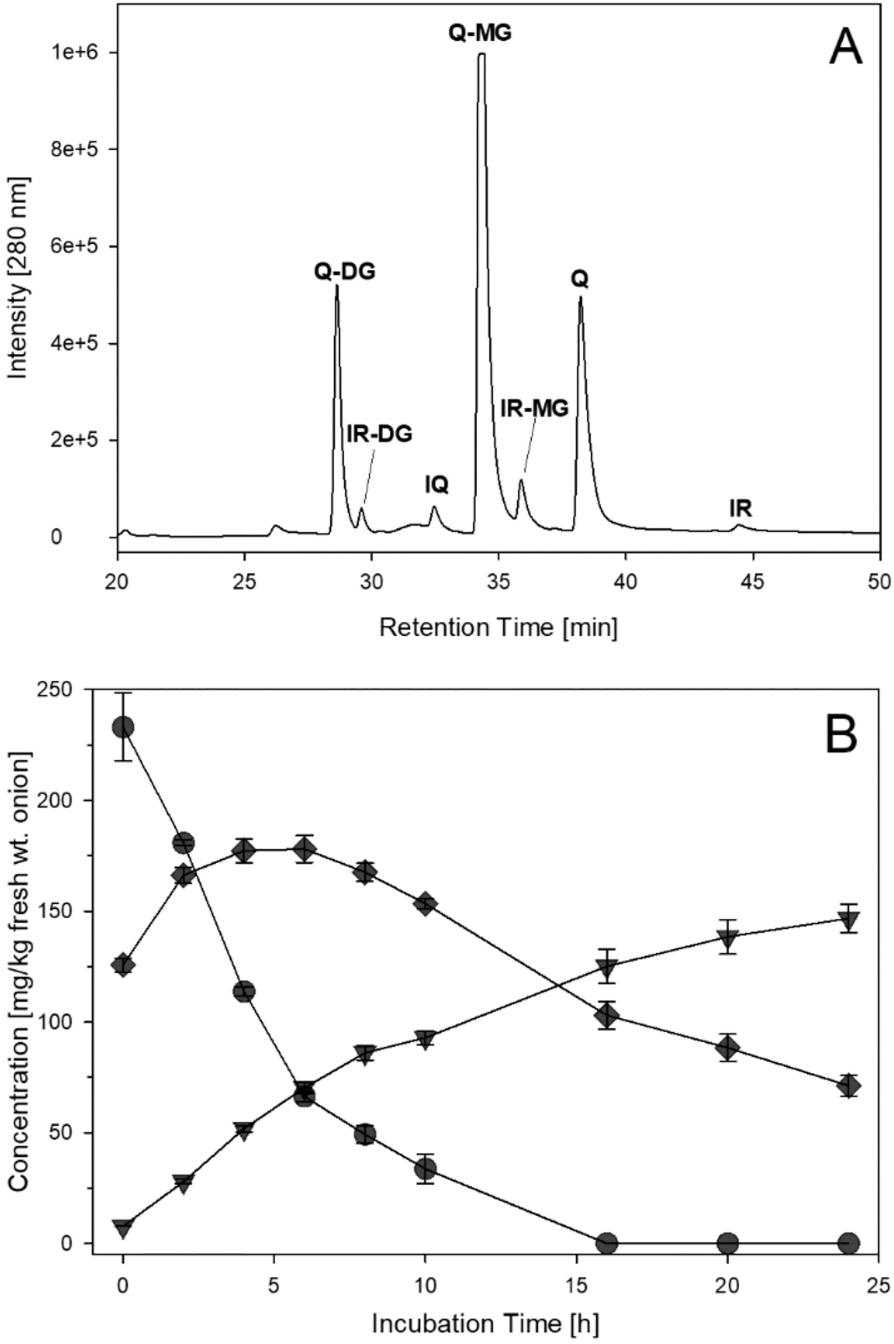
(A) HPLC-UV-Chromatogram of minced onion after 6 h of incubation (37 °C, aeration, exclusion of light), Q-DG: quercetin-3,4′-*O*-diglucoside, IR-DG: isorhamnetin-3,4′-*O*-diglucoside; IQ: isoquercitrin; Q-MG: quercetin-4′-*O*-monoglucoside; IR-MG: isorhamnetin-4′-*O*-monoglucoside; Q: quercetin; IR: isorhamnetin. (B) Release of quercetin (▼) from quercetin-3,4′-*O*-diglucosid (●) and quercetin-4′-*O*-monoglucosid (⧫).

**Figure 7:**
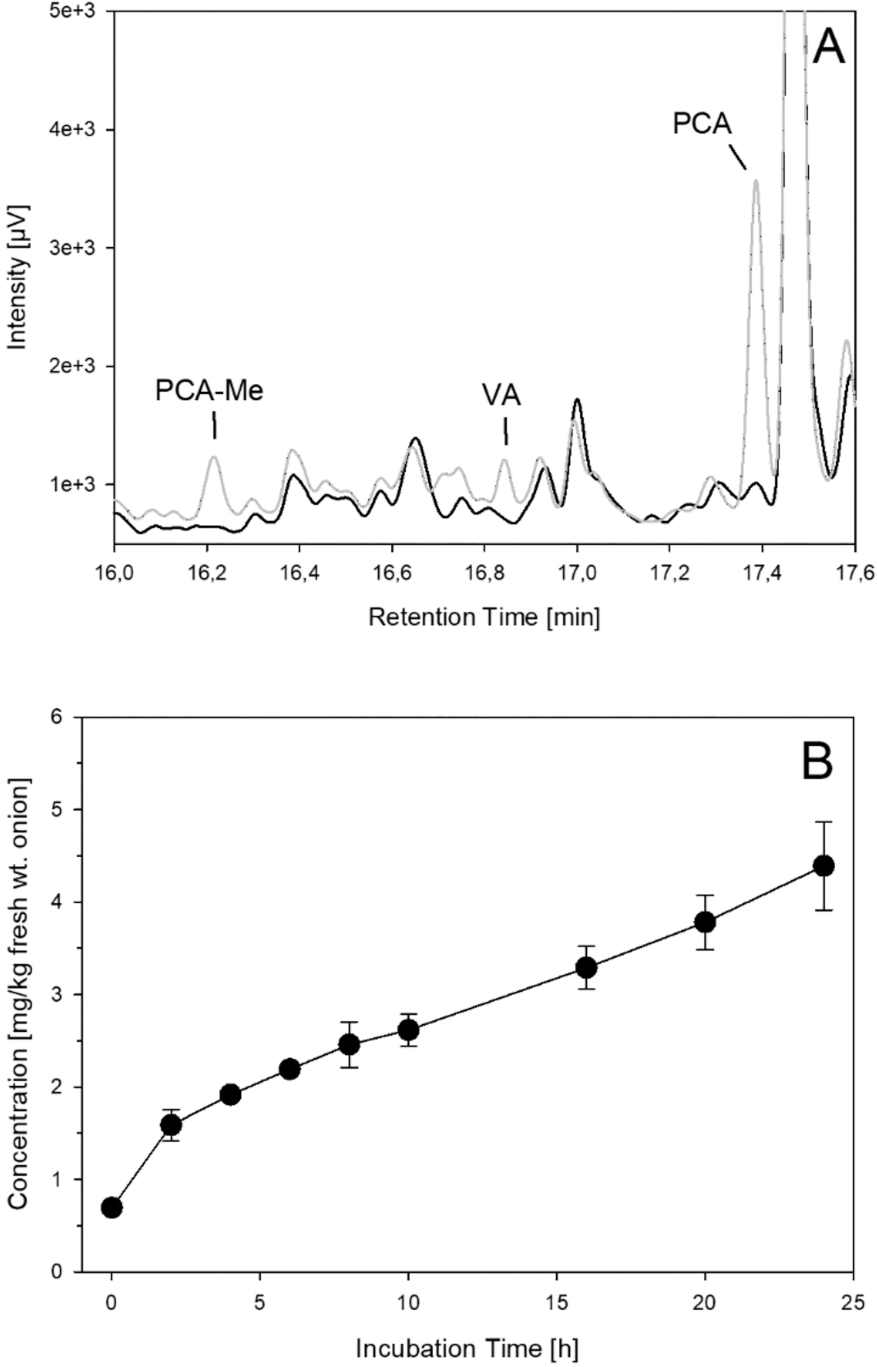
(A): GC-MS-chromatogram of minced onion incubations (37 °C, aeration, exclusion of light, workup with trimethylsilylation) after 24 h (gray) compared to start of incubation (black), PCA-ME: protocatechuic acid methyl ester; VA: vanillic acid; PCA; protocatechuic acid. (B) Increase the sum of protocatechuic acid and methyl ester (●).

### Singlet Oxygen-Induced Flavonol Degradation in Leek Samples

To further extend the importance of singlet oxygen-triggered flavonol degradation, leek samples were minced and incubated as above. Leek contains significant amounts of kaempferol glycosides (up to 56 mg/kg fresh wt.).^21^ Quercetin concentrations in leek were reported contradictory ranging from absence to significant quantities (up to 310 mg/kg fresh wt.).^21,33^ However, in the present investigation, neither quercetin nor the respective glycosides were ever detected in the commercial leek samples used. The quantitatively important kaempferol species are various 3-*O*-mono- and diglycosides partially esterified with malonic and cinnamic acids.^34,35^ This should lead to the release of *p*-coumaric, ferulic, and caffeic acid during processing-induced hydrolysis. In the present study, LC-MS screening of minced leek incubations verified that, other than kaempferol, only ferulic acid together with *p*-hydroxybenzoic acid as the anticipated, singlet oxygen triggered benzoic acid from kaempferol fragmentation was formed (Figure 8). Neither kaempferol nor ferulic acid can generate singlet oxygen due to their phenolic constitution (Table 1). However, gallic acid as a strong singlet oxygen source was quantitated to accumulate slowly during the 24 h incubation period up to 0.5 mg/kg fresh wt. Gallic acid has been found in leek in considerable amounts up to 67 mg/kg dry wt. only after acid hydrolysis (given a typical water content of 85%, this translates to about 10 mg/kg fresh wt.), which has to be attributed to the presence of hydrolyzable tannins.^36^ However, as already mentioned above information for specific qualitative and quantitative phenolic content of leek is scarce and inconsistent also quoting gallic acid concentrations without acid hydrolysis up to 757 mg/kg dry wt.^37^ In contrast, the amounts analyzed herein were rather low but clearly increased during incubation, which must be explained by nonenzymatic slow hydrolysis of gallotannins under the very weak acidic pH in shredded leek. Similar observations for tannin hydrolysis were made by Rakshit and Srivastav.^38^ Incubations under deaeration gave similar levels of gallic acid release, while the production of *p*-hydroxybenzoic acid was significantly lower. Thus, the formation of *p*-hydroxybenzoic acid was assigned to the singlet oxygen produced by gallic acid to induce the fragmentation of kaempferol. To further highlight this relationship, leek was incubated with a gallic acid addition of 42.5 mg/kg fresh wt. under readjustment from pH 5.3 to 6.3 of native leek incubations and a strong increase of *p*-hydroxybenzoic acid up to 7.5 mg/kg fresh wt. was verified.

**Figure 8:**
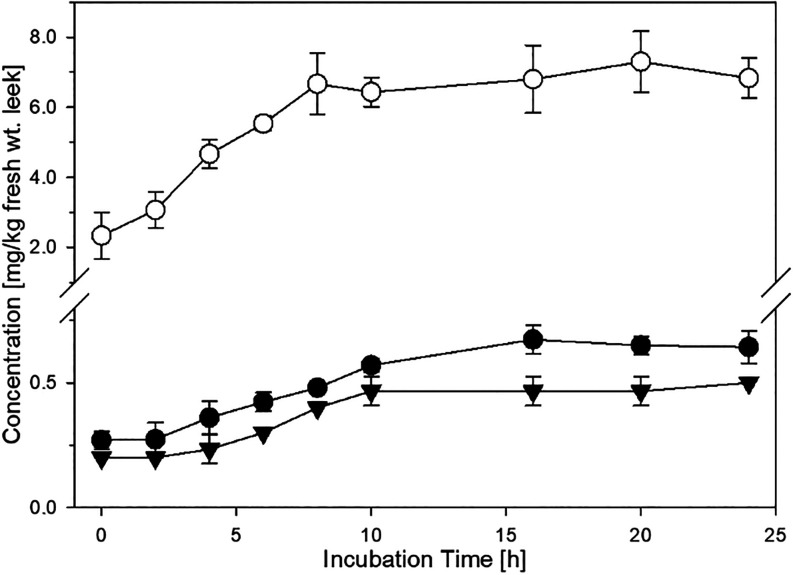
Generation of *p*-hydroxybenzoic acid (HBA) and release of gallic acid (GA) in minced leek incubations (37 °C, aeration, exclusion of light); HBA (●) and GA (**▼**) in native leek; HBA (○) in leek spiked with 42.5 mg/kg fresh wt. GA.

In summary, singlet oxygen generation and follow-up reactions were established under typical food processing conditions in an aqueous milieu at neutral pH in the absence of light. Plant phenolics of all three major groups, hydroxybenzoic acids, cinnamic acids, and flavonoids, bearing catechol or gallo di- or trihydroxylation motives, were potent singlet oxygen sources. Model reactions showed that such induced fragmentation of flavonols to give hydroxybenzoic acids is a major outcome to reach up to 25 mol %. Investigation of onions and leek verified for the first time that singlet oxygen-triggered fragmentation indeed proceeds in complex food matrices. As the present investigation is totally different from the established photochemically induced singlet oxygen approaches, this will now trigger research including not only other phytophenolic structures but also other major food constituents.

## Supplementary Material



## Notes

Phloridzin fragmentation to *p*-dihydrocoumaric acid as a selective probe for singlet oxygen. Isolation and characterization of methanol flavanone adduct **3**: 2,7-dimethoxy-3,3,5,3′,4′-pentahydroxyflavanone (Figure SI-1); (A) HPLC-UV chromatogram of the crude reaction mixture (0.5 mM quercetin and 2 mM gallic acid in H_2_O/methanol) at 280 nm; (B) CID of adduct **2**; (C) CID of adduct **3** (Figure SI-2); ^1^H NMR: reaction mixture **2 + 3** isolated from methanol solutions in DMSO-d6; ^2^H NMR: isolated reaction mixture **2 + 3** taken up in CD_3_OD, solvents evaporated and measure in nondeuterated DMSO (Figure SI-3); high-resolution mass and ^1^H- and ^13^C NMR spectroscopic data of 2,7-dimethoxy-3,3,5,3′,4′-pentahydroxyflavanone **3** (in DMSO-d6) (Table SI-1); ^1^H NMR of isolated mixture **2 + 3** in DMSO-d6 (Figure SI-4); ^13^C NMR of isolated mixture **2 + 3** in DMSO-d6 (Figure SI-5); isolated mixture **2 + 3**: Selected HMBC-NMR correlations verifying 7-OCH_3_ in adduct **3** (in DMSO-d6) (Figure SI-6); proposed mechanism for equilibrium between adduct **2** and adduct **3** (Figure SI-7) (PDF)

## Abbreviations

DMN: dimethylnaphthalene
DMN-EP: dimethylnaphthalene-endoperoxide
GA: gallic acid
IR: isorhamnetin
IR-MG: isorhamnetin-4′-*O*-monoglucoside
IR-DG: isorhamnetin-3,4′-*O*-diglucoside
PCA: protocatechuic acid
PCA-ME: protocatechuic acid methyl ester
*p*-HB: *para*-hydroxybenzoic acid
Q: quercetin
IQ: isoquercitrin
Q-MG: quercetin-4′-*O*-monoglucoside
Q-DG: quercetin-3,4′-*O*-diglucoside
VA: vanillic acid
