# Supplementary material for: Singlet Oxygen Generated by Quercetin and Gallic Acid Leads to Oxidative Fragmentation of Flavonols in Onions and Leek
Source: J Agric Food Chem. 2026 Feb 5;74(6):5640–50. doi: 10.1021/acs.jafc.5c14934 (PMC12921883; doi:10.1021/acs.jafc.5c14934)

SUPPORTING INFORMATION

Singlet oxygen generated by quercetin and gallic acid leads to oxidative fragmentation of flavonols in onions and leek

Vanessa K. Fokuhl[1], Lea M. Kahl[1], Niels Heise[2] and Marcus A. Glomb[1]*

[1]Institute of Chemistry, Food Chemistry, Martin-Luther-University Halle-Wittenberg, Kurt-Mothes-Str. 2, 06120 Halle/Saale, Germany

[2]Institute of Chemistry, Organic Chemistry, Martin-Luther-University Halle-Wittenberg, Kurt-Mothes-Str. 2, 06120 Halle/Saale, Germany

*To whom correspondence should be addressed (e-mail marcus.glomb@chemie.uni-halle.de, Fax ++049-345-5527341)

Figure SI-1:

Phloridzin fragmentation to *p*-dihydrocoumaric acid as a selective probe for singlet oxygen

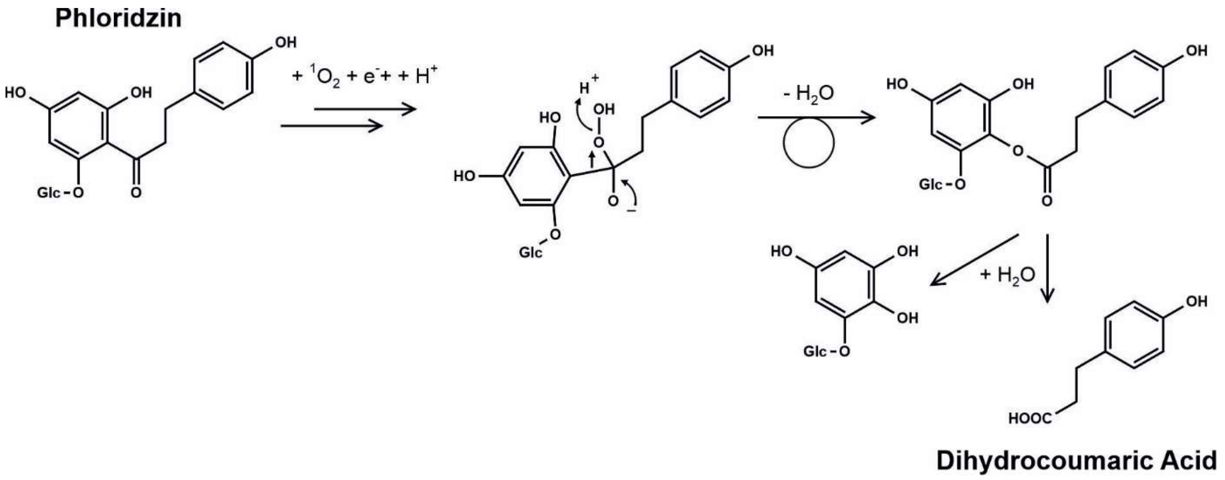

Isolation and Characterization of Methanol Flavanone Adduct 3: 2,7-Dimethoxy-3,3,5,3',4'-pentahydroxy-flavanone.

Quercetin methanol adduct **3** was isolated together with adduct **2** from 4 L of an upscaled reaction mixture of 0.5 mM quercetin with 2 mM gallic acid in a H_2O/methanol (1/1, v/v) mixture by a combination of flash chromatography and preparative RP-18 chromatography. **Figure SI-2** shows the crude reaction mixture with two badly resolved adduct peaks and their respective CID-spectra. These revealed an increase of *m/z* 14 for the molecular mass of **3** [364] in comparison to **2** [350], but otherwise a very similar fragmentation pattern. The bad peak resolution together with the in between tailing of both peaks already implied a pair of 2 compounds in an equilibrium. Indeed, a separate isolation was not possible. Enriched fractions from the aqueous methanol incubation with chromatographic eluents using methanol always returned to the original equilibrium ratio visible in **Figure SI-2 a**, estimated at 1 to 2 for **3**/**2**. Indeed, when **3** was taken up in ethanol and the solvent evaporated, the molecular mass difference of this adduct now moved to *m/z* 28 compared to **2** and showed that only one of the two methoxy groups was exchangeable to an ethoxy group. This mechanistic relationship was used to locate the position of the second implementation of a methoxy group in **3** by the use of deuterated methanol (**Figure SI-3**). ^{2}H-NMR in comparison to ^{1}H-NMR verified only one resonance at about 3.8 ppm, significantly low field from the stable bound methoxy group at 2.9 ppm in ^{1}H-NMR.

Unequivocal structure elucidation by ^{1}H-, ^{13}C-, HSQC- and HMBC-NMR together with confirmation of the elemental composition by HRMS led to the structure assignment depicted in **Table SI-1** as 2,7-dimethoxy-3,3,5,3′,4′-pentahydroxy-flavanone **3**. Most resonances in ^{1}H-NMR and ^{13}C-NMR were virtually identical to **2**, except for position 7, 8, 9 and the extra methoxy group (**Figure SI-4 and SI-5**). Above equilibrium ratio of **3** to **2** was confirmed by comparing proton signals for H-8 at 5.83 ppm (**3**) to 5.99 ppm (**2**), but also for the methoxy groups at 3.83 ppm (**3**) with peak area at 2.94 ppm (**2**+**3**). Specific confirmation of the implementation of the methoxy group at position C-7 in **3** was verified by HMBC-NMR as shown in **Figure SI-6**. Protons at H-12 correlated to C7, while proton at H-8 showed crosspeaks to C-7, C-9 and C-5. Given the still at the C-3 located hydrate (90.7 ppm) in **3**, the reversible exchange of alcohols at C-7 might be unexpected at the first sight. This might also explain the false interpretation of HRMS data of irradiated flavonol-methanol reaction mixtures to tentatively assign the methanol adduct formation to a hemiacetal formation at C-3.[30] On the other hand as shown in **Figure SI-7** the position C-7 is highly activated by the two carbonyl functions at C-3 and C-4, which plausibly explains the easy incorporation of methanol at C-7.

Figure SI-2:

(A) HPLC-UV chromatogram of the crude reaction mixture (0.5 mM quercetin and 2 mM gallic acid in H_2O/methanol) at 280 nm; (B) CID of adduct **2**; (C) CID of adduct **3**.

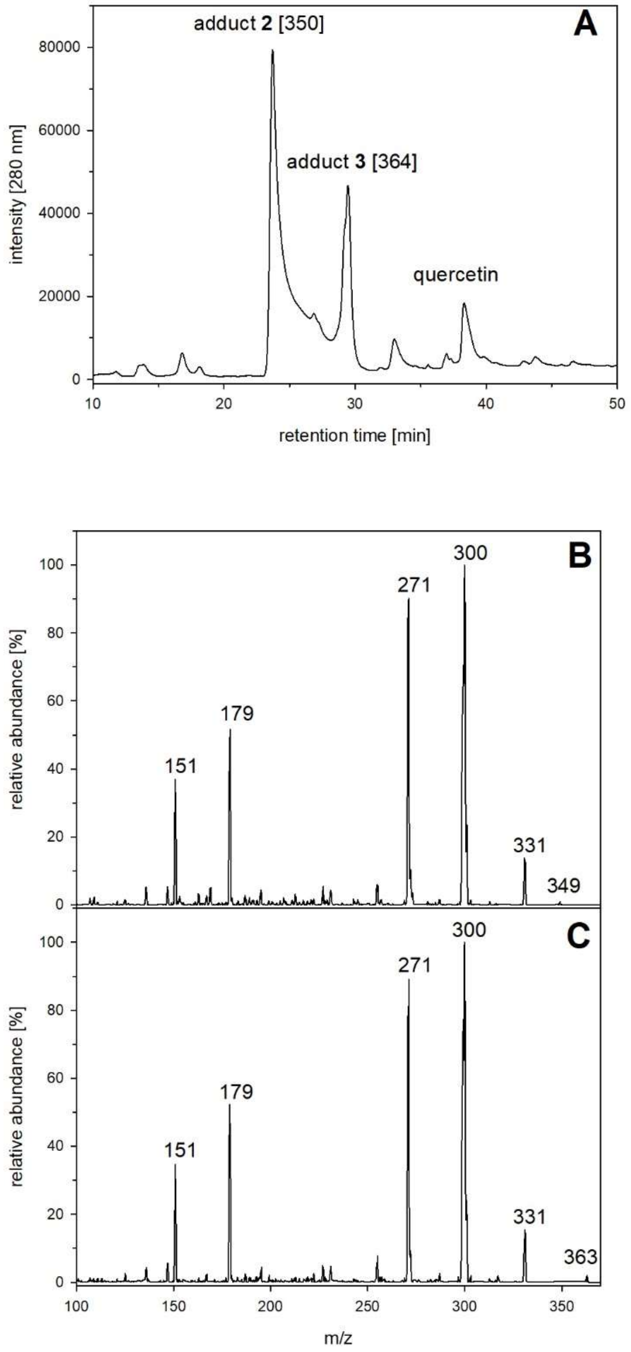

Figure SI-3.

^{1}H-NMR: reaction mixture **2**+**3** isolated from methanol solutions in DMSO-*d6*

^{2}H-NMR: isolated reaction mixture **2**+**3** taken up in CD_3OD, solvents evaporated and measured in non-deuterated DMSO.

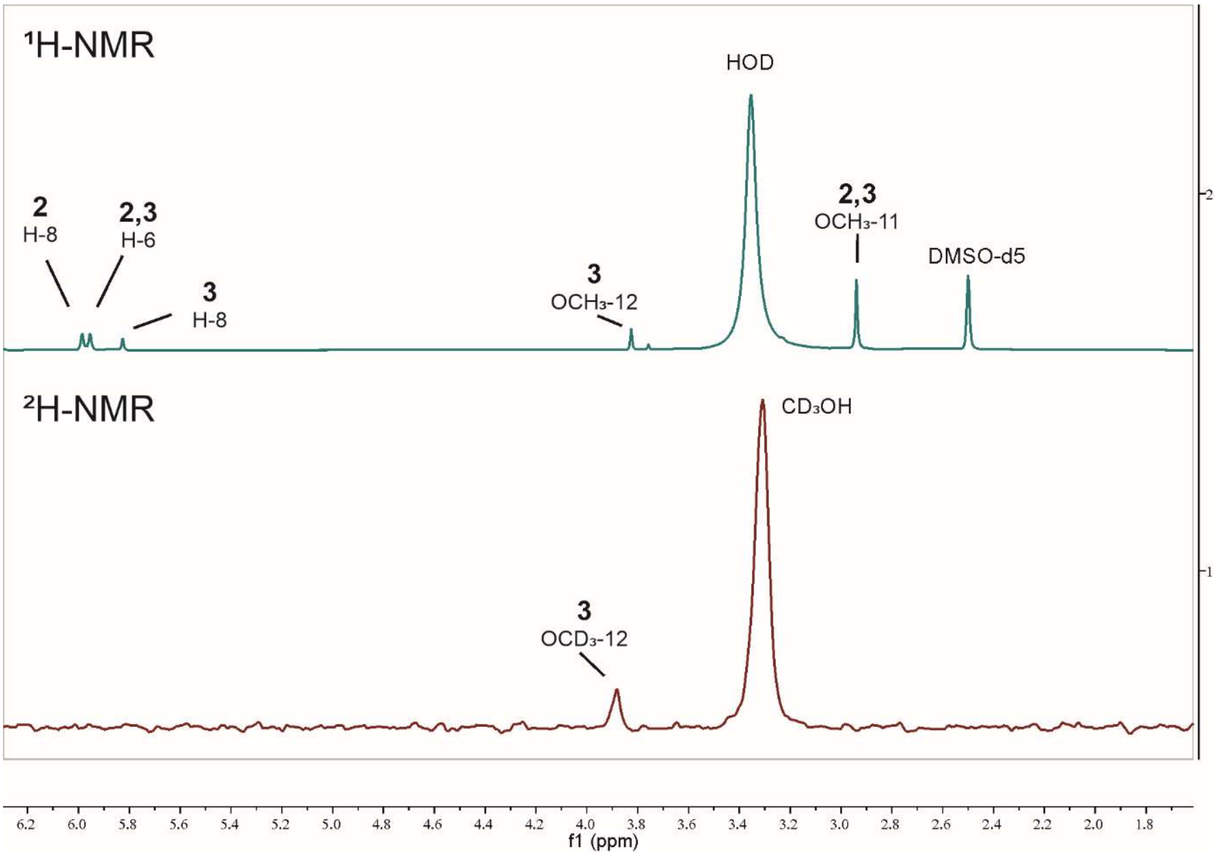

Table SI-1. High-Resolution Mass and ^{1}H- and ^{13}C-NMR Spectroscopic Data of 2,7-Dimethoxy-3,3,5,3′,4′-pentahydroxy-flavanone **3** (in DMSO-*d6)*.

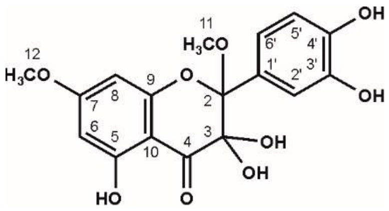

**HR-MS [M-H]$^{-}$ (*m/z*)**		363.0715
**calcd. $C_{17}H_{15}O_9^{-}$ (*m/z*)**		363.0722
**C/H**	δ ^{1}H [ppm]	δ ^{13}C [ppm]
**2**	-	106.8
**3**	-	90.7
**4**	-	194.7
**5**	-	163.7
**6**	5.95 (s, 1H)	96.3
**7**	-	170.3
**8**	5.83 (s, 1H)	95.1
**9**	-	161.9
**10**	-	99.7
**11**	2.94 (s, 3H)	50.1
**12**	3.83 (s, 3H)	52.0
**1‘**	-	124.1
**2‘**	7.03 (d, 1H)	116.8
	^{4}J = 2.1 Hz	
**3‘**	-	144.1
**4‘**	-	145.8
**5‘**	6.74 (d, 1H)	114.4
	^{3}J = 8.2 Hz	
**6‘**	6.87 (dd, 1H)	120.3
	^{3}J = 8.2 Hz	
	^{4}J = 2.1 Hz	

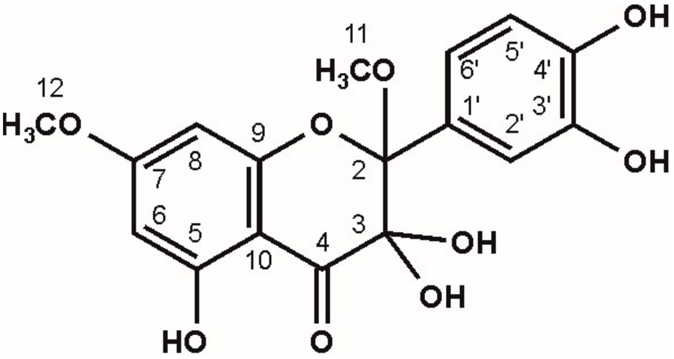

Figure SI-4.

^{1}H-NMR of isolated mixture **2** + **3** in DMSO-*d6*

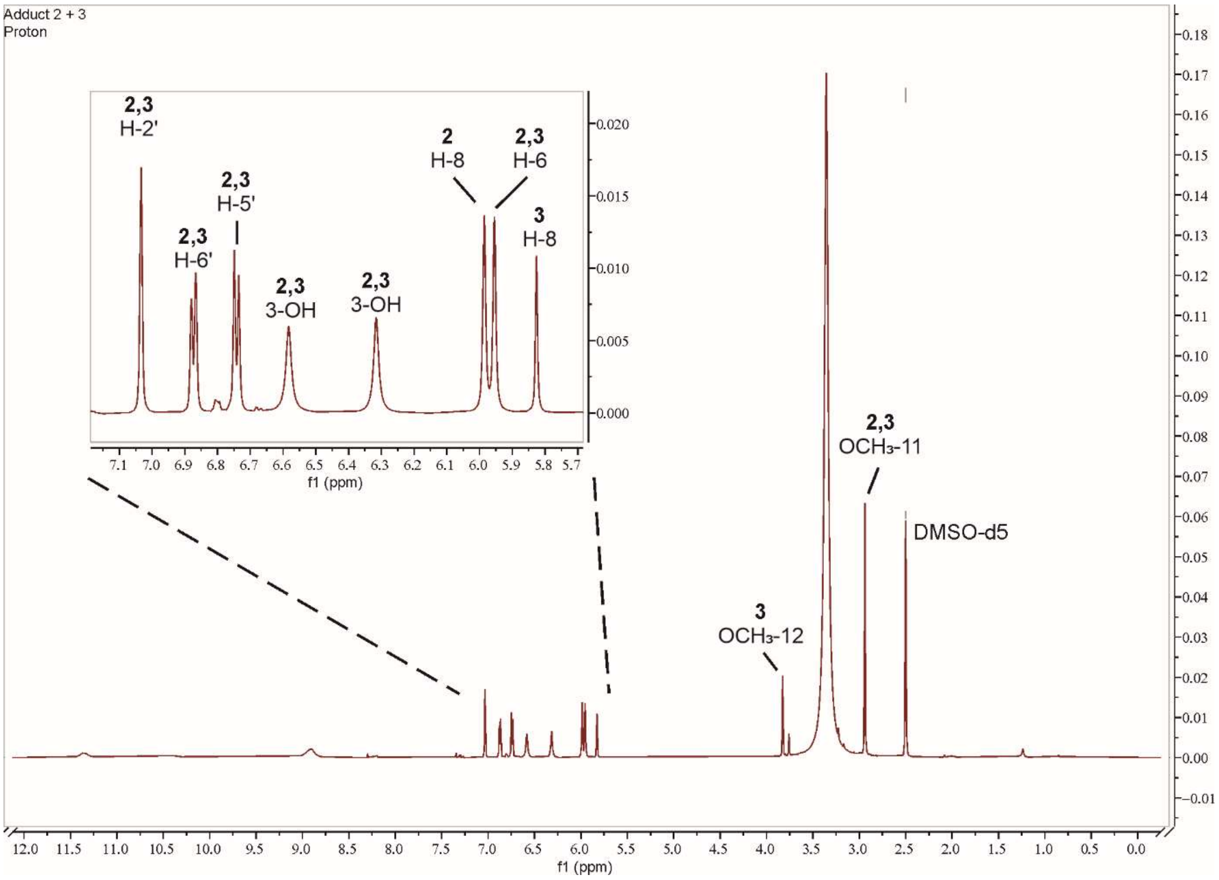

Figure SI-5.

^{13}C-NMR of isolated mixture **2** + **3** in DMSO-*d6*

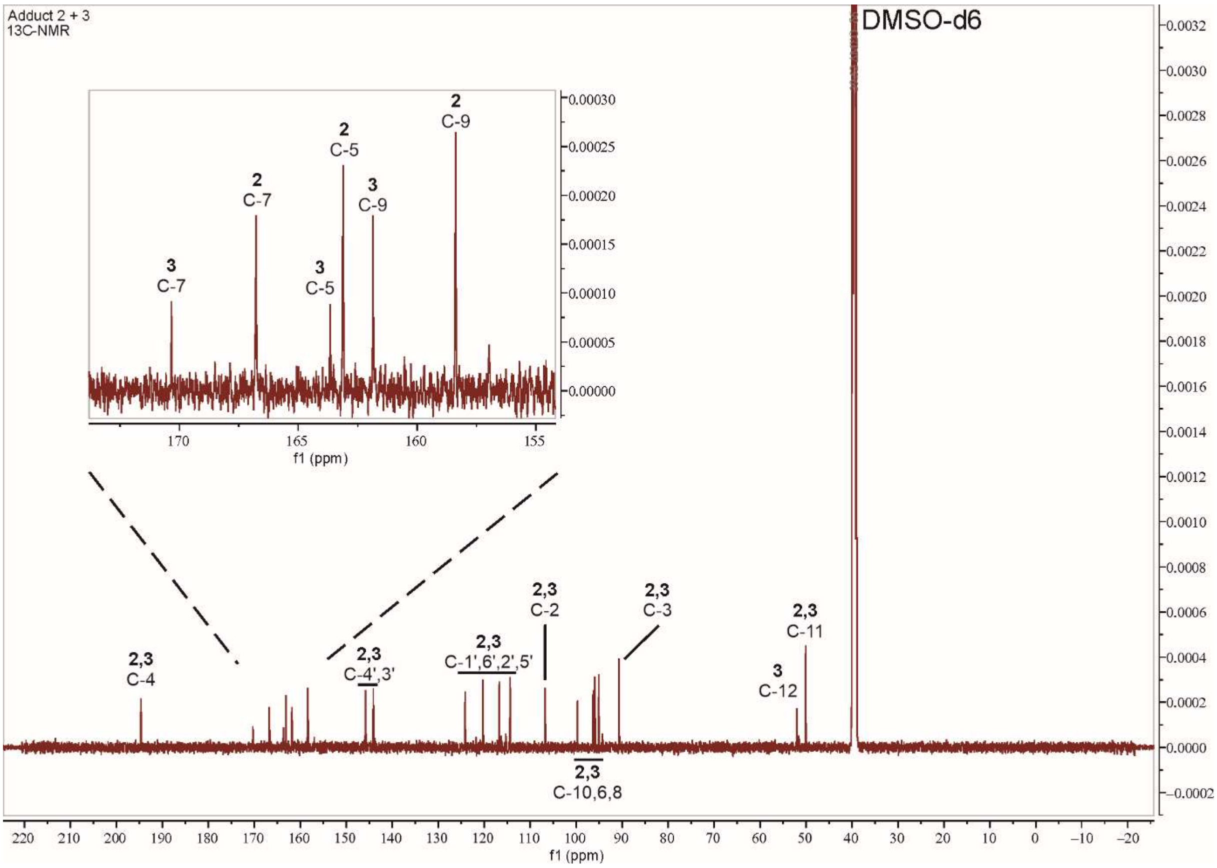

Figure SI-6.

Isolated mixture **2** + **3**: Selected HMBC-NMR correlations verifying 7-OCH_3 in adduct **3** (in DMSO-*d6*)

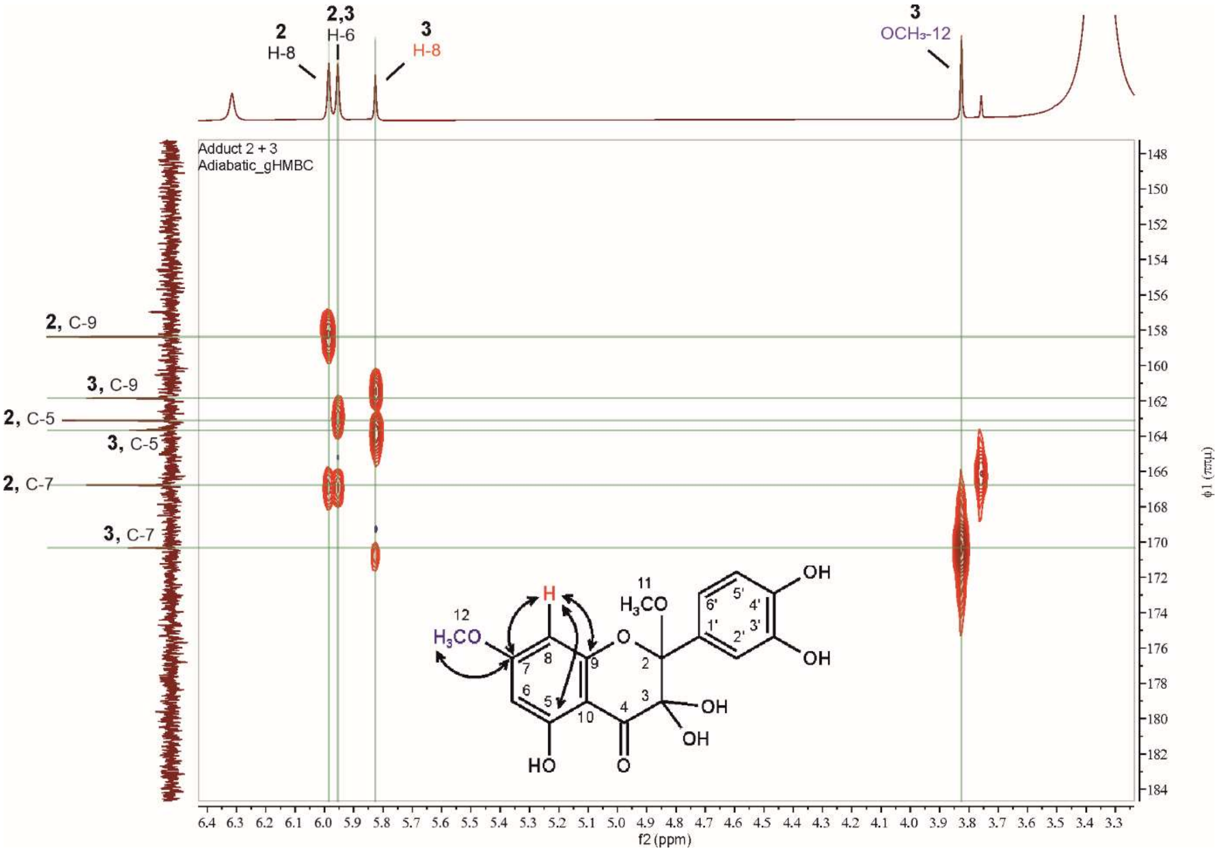

Figure SI-7.

Proposed mechanism for equilibrium between adduct **2** and adduct **3**.

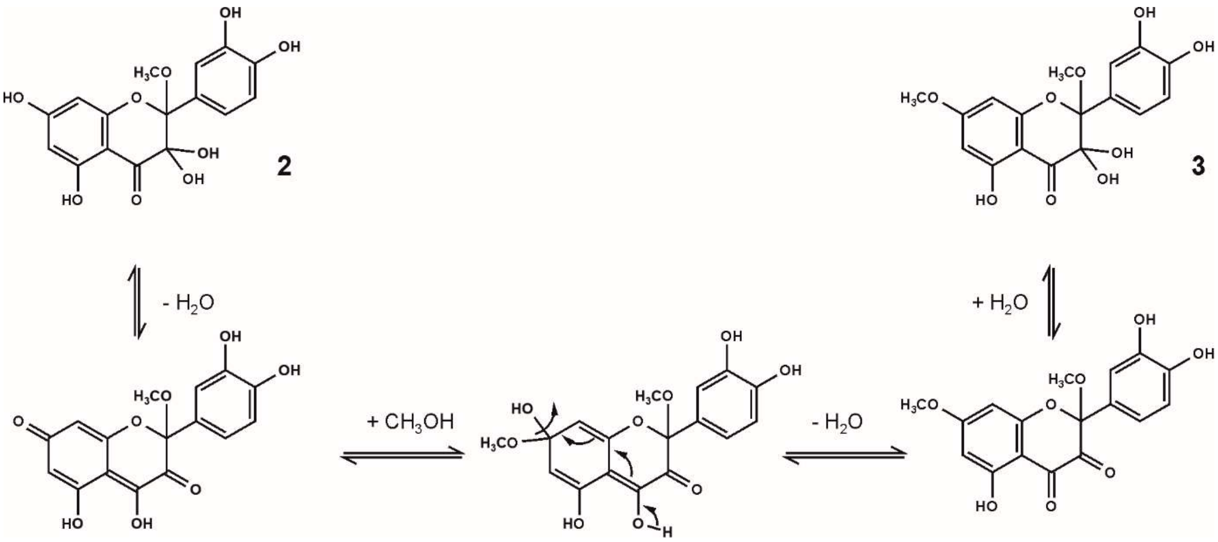

Supplement: Supplementary file 1 [file jf5c14934_si_001.pdf]
